# In the blood: biomarkers for amyloid pathology and neurodegeneration in Alzheimer’s disease

**DOI:** 10.1093/braincomms/fcaa054

**Published:** 2020-07-02

**Authors:** Jamie Toombs, Henrik Zetterberg

**Affiliations:** f1 Centre for Discovery Brain Sciences, UK Dementia Research Institute, The University of Edinburgh, UK; f2 UK Dementia Research Institute at UCL, London, UK; f3 Department of Neurodegenerative Disease, UCL Institute of Neurology, London, UK; f4 Department of Psychiatry and Neurochemistry, Institute of Neuroscience and Physiology, the Sahlgrenska Academy at the University of Gothenburg, Mölndal, Sweden; f5 Clinical Neurochemistry Laboratory, Sahlgrenska University Hospital, Mölndal, Sweden

## Abstract

This scientific commentary refers to ‘Plasma total-tau, neurofilament light chain and amyloid-β levels and risk of dementia: a population-based study’ by de Wolf *et al.* (https://doi.org/10.1093/brain/awaa054), and ‘Relationship of amyloid-b1–42 in blood and brain amyloid: Ginkgo Evaluation of Memory Study’ by Lopez *et al.* (https://doi.org/10.1093/braincomms/fcz038), two papers that illustrate these latest developments.

**This scientific commentary refers to ‘Plasma total-tau, neurofilament light chain and amyloid-β levels and risk of dementia: a population-based study’ by de Wolf *et al.* (https://doi.org/10.1093/brain/awaa054), and ‘Relationship of amyloid-b1–42 in blood and brain amyloid: Ginkgo Evaluation of Memory Study’ by Lopez *et al.* (https://doi.org/10.1093/braincomms/fcz038), two papers that illustrate these latest developments**.

## Introduction

Among the most impactful and fast developing aspects in neurodegeneration research and clinical practice over the last 30 years have been the development of biomarkers. A biomarker is a measurable indicator of a biological state or pathological condition (Zetterberg, 2019). Frustratingly, the field finds itself in tension between the increasing power and role of biomarkers to detect and predict the pathologies that underlie dementia versus stagnation in prophylactic, therapeutic or reparative interventions. Reduction in the cost of trials by cheap, accurate and accessible pre-screening and better understanding of how biomarkers relate to brain pathology are important to resolve this problem. Here, we discuss the role blood-based biomarkers can play in the context of two recent papers by de Wolf *et al.* (2020) and Lopez *et al.* (2020).

## Blood biomarkers in context

Both papers have emerged in the context of a burgeoning paradigm shift in the potential of fluid biomarkers. Over the past decade, cerebrospinal fluid (CSF) and positron emission tomography (PET) biomarkers have dominated neurodegeneration research and guided drug design. CSF amyloid beta 1-42 (Aβ42), total tau (T-tau) and phosphorylated tau_181_ (P-tau_181_) and ^11^C Pittsburgh Compound B, florbetapir and florbetaben PET for Aβ pathology are now well validated for Alzheimer’s disease with 85–95% sensitivity and specificity (Zetterberg and Bendlin, 2020). Important recent developments are the introduction of certified reference methods and materials for Aβ42, and second-generation tau PET. However, collection of CSF is sometimes regarded as a minor surgical procedure, requiring specialist training, and brain imaging techniques are costly, also require specialist training, and employ radioactive tracers in the case of PET. These issues limit scalable testing and who can access them.

**Figure 1:**
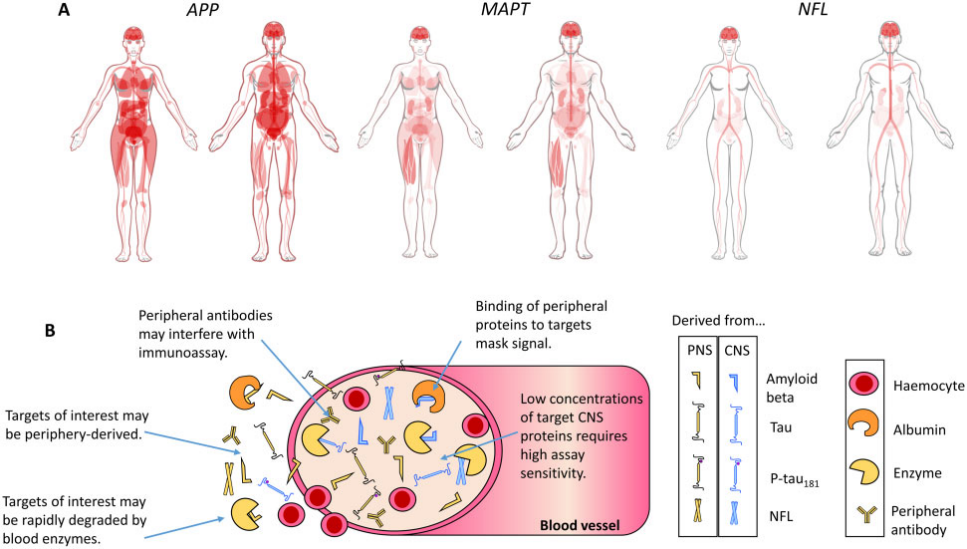
**Caveats for blood biomarkers.** Measurement of brain-derived proteins in blood is complicated by a number of factors. (**A**) Tissue expression of genes that encode proteins relevant to AD. Proteolysis of amyloid precursor protein (APP), encoded by the *APP* gene, generates Aβ. Microtubule-associated protein tau (*MAPT*) encodes tau protein. Neurofilament light chain (NFL) encodes the protein of the same name. Diagrams for APP (www.proteinatlas.org/ENSG00000142192-APP/tissue), MAPT (www.proteinatlas.org/ENSG00000186868-MAPT/tissue), and NFL (www.proteinatlas.org/ENSG00000277586-NEFL/tissue) generated by Human Protein Atlas (Uhlen *et al*., 2015). Red indicates gene expression. From the expression patterns, it is clear that NFL is the most neuronal-specific of the biomarkers, which may explain why its plasma concentration associates the best with neurodegeneration. (**B**) A simplistic diagram of brain biomarkers in blood, highlighting the key issues for accurate detection.

Blood-based biomarkers have the potential to circumvent or diminish many of these limitations. Phlebotomy is a comparatively cheap, routine and un-invasive procedure, and so fluid biomarker analysis from blood is highly scalable. Much scepticism surrounded early data from blood because of poor reproducibility (reasons portrayed in Fig. 1). Recent improvements in instrument sensitivity have rapidly begun to change the state of play. Sub-femtomolar concentration detection afforded by single molecule array (Simoa) technology, and improvements in immunoprecipitation mass spectrometry platforms enable accurate, consistent and high sensitivity measurement even after the extensive dilution of plasma and serum.

## Amyloid beta

Of candidate biomarkers in plasma, Aβ42 and Aβ40 have been the most extensively studied (Olsson *et al.*, 2016). Many early studies showed no change or even increases in blood Aβ in Alzheimer’s disease versus control samples (Olsson *et al.*, 2016); however, recent work using high sensitivity assays have begun to show the expected decrease and correlation between CSF and plasma Aβ42/40 ratios (Janelidze *et al.*, 2016; Ovod *et al.*, 2017; Nakamura *et al.*, 2018).

The work of de Wolf *et al.* contributes one of the largest longitudinal analyses of plasma Aβ42 and Aβ40 to date. Measured by Simoa, baseline Aβ42 and Aβ42/40 levels, but not Aβ40, were predictive of conversion to dementia. Hazard ratios (HR) showed that low plasma Aβ42 was significantly associated with conversion to all-cause dementia and Alzheimer’s disease. Dividing the dataset into quartile groups delineated the relationship between lower Aβ42 and increased risk further, and showed that the association was stable over time.

Trajectory analysis demonstrated that over 13 years Aβ42 declined at similar rates in both Alzheimer’s disease and non-Alzheimer’s disease converters. Aβ42/40 was significantly altered over time in Alzheimer’s disease converters, but the effect size was negligible and driven by Aβ40. Similarly, Lopez *et al.* found that changes in Aβ42 over an eight year period were marginal and unrelated to brain amyloid deposition. The mean age in both studies was relatively advanced [71.9 years (de Wolf *et al.*, 2020) and 85 years (Lopez *et al.*, 2020)], and it may be that samples were taken too late to catch longitudinal intra-individual Aβ changes. It must also be noted that Lopez *et al.* excluded dementia converters from their analysis. Nevertheless, this raises the important question of what Aβ measured from blood means biologically. Plasma Aβ is influenced by a number of factors (Fig. 1). If plasma Aβ concentrations are periphery driven, whether or not they are predictive of dementia, it would be difficult to use them to make inferences about, or therapeutically target, processes occurring in the brain. It would also raise questions about the extent to which dementia arises from CNS pathology in relative isolation.

Results from Lopez *et al.* showed that ^11^C-PiB PET positivity (measured in 2009) correlated significantly with decreased plasma Aβ42 (measured by enzyme-linked immunosorbent assay) in individuals whose blood was collected in the 2000–02 group of their study. All brain regions studied, particularly the anterior cingulate gyrus and frontal cortex, were significant contributors. However, the story was not straight-forward and data from the 2008–09 group only showed a non-significant tendency for correlation of plasma Aβ with brain amyloid deposition, with an inconsistent pattern of regional contribution. Other studies have identified correlation between plasma Aβ42/40 and PET-based amyloid deposition (Janelidze *et al.*, 2016; Nakamura *et al.*, 2018), as well as CSF concentrations (Janelidze *et al.*, 2016). Thus, whilst brain reservoir likely contributes to plasma Aβ42 concentrations, the relationship of plasma Aβ to neurodegenerative pathology remains in question, especially if not measured by the latest ultra-sensitive techniques.

## Neurofilament light chain

Plasma/serum concentrations of NFL correlate well with CSF (Zetterberg *et al.*, 2016) and have been among the most consistent blood markers for all-cause neurodegeneration (Mattsson *et al.*, 2019; Janelidze *et al.*, 2020; Thijssen *et al.*, 2020). Data presented by de Wolf *et al.* add more positive data to this story. Baseline plasma NFL concentration was strongly predictive of conversion to all-cause dementia, and particularly Alzheimer’s disease and vascular dementia. Quartile analysis highlighted this pattern further and when the highest and lowest quartiles of Aβ42 and NFL concentration were combined and compared, HRs increased substantially (all-dementia HR: 9.5, Alzheimer’s disease HR: 15.7). Of particular interest, cumulative incidence analysis showed that plasma NFL concentration increased 3.4 times faster in participants who developed Alzheimer’s disease versus no dementia. These changes were detectable 9.6 years before Alzheimer’s disease diagnosis.

Results from a single biomarker test must be interpreted in relation to cross-sectionally determined cut-points from sample populations, which may or may not have high relevance to the individual. Multiple measurements taken longitudinally from the same individual would provide an internal reference point and thus circumvent such issues. If pre-symptomatic disease-associated changes in a blood-based biomarker, such as NFL, could be detected over a clinically relevant time interval this could be a powerful tool. A decade may not be such a time frame, however some data suggest that plasma NFL changes may track certain aspects of underlying pathology over 15–30 months (Mielke *et al.*, 2019).

Lopez *et al.* did not analyse the relationship between NFL and brain pathology, but other recent studies have addressed this question. Despite finding elevated plasma NFL in cortical–basal syndrome, progressive supranuclear palsy, behavioural variant frontotemporal dementia and Alzheimer’s disease compared to healthy controls, Thijssen *et al.* (2020) found that plasma NFL was not related to either tau PET (flortaucipir) or Aβ PET (PiB/florbetapir). Mielke *et al.* observed a similar relationship between baseline NFL concentration, PiB PET and fluorodeoxyglucose (FDG)-PET (a biomarker for synaptic dysfunction/degeneration) (Mielke *et al.*, 2019). However, longitudinal elevation of plasma NFL were associated declines in hippocampal volume, cortical thickness, FDG-PET, corpus callosum fractional anisotropy and global cognitive z scores, as well as with increasing amyloid PET positivity (Mielke *et al.*, 2019). In summary, it is not yet clear exactly how well peripherally measured NFL reflects CNS neurodegeneration (see also Fig. 1), but indications are that brain reservoir has a net contribution over time.

## Tau

Recent tau kinetics data suggest that Alzheimer’s disease-related increase of CSF T-tau and P-tau may be a neuronal response to Aβ pathology (Sato *et al.*, 2018), rather than a direct reflection of neurodegeneration and tangle pathology. Despite this, de Wolf *et al.* found plasma T-tau to be unchanged in Alzheimer’s disease versus cognitively normal individuals, consistent with the observations of Verberk *et al.* (2018), but in contrast to others (Mattsson *et al.*, 2016; Olsson *et al.*, 2016; Mielke *et al.*, 2018; Pase *et al.*, 2019). Such conflicting evidence likely stem from the poor correlation of plasma T-tau with CSF T-tau (Mattsson *et al.*, 2016), and factors portrayed in Fig. 1.

A notable gap in the work of de Wolf and Lopez *et al.* is that of plasma P-tau_181_. CSF P-tau_181_ is a core Alzheimer’s disease-specific biomarker, found to be increased relatively early in the disease course, potentially as part of a neuronal response to Aβ pathology. In four recent studies, baseline plasma P-tau_181_ was significantly increased, associated specifically with conversion of cognitively normal individuals to Alzheimer’s disease (Mielke *et al.*, 2019; Janelidze *et al.*, 2020; Karikari *et al.*, 2020; Thijssen *et al.*, 2020). Plasma P-tau_181_ accurately discriminated Alzheimer’s disease dementia from non- Alzheimer’s disease neurodegenerative diseases with sensitivity and specificity similar to CSF Aβ42/Aβ40 and CSF T-tau, and slightly worse than CSF P-tau181 combined with Tau PET. As a point of comparison with NFL data from de Wolf *et al.*, a longitudinal study found that baseline plasma P-tau_181_ concentration was a significant predictor of Alzheimer’s disease conversion (Janelidze *et al.*, 2020). When data were thresholded based on a 1.81 pg ml^–1^ cut-point, associated risk increased markedly (HR = 10.9) for those with higher versus lower concentrations (Janelidze *et al.*, 2020).

Plasma P-tau_181_ correlates well with CSF P-tau_181_, as well as both Aβ PET and tau PET (Mielke *et al.*, 2019; Thijssen *et al.*, 2020). Furthermore, plasma P-tau_181_ has been shown to be significantly associated with Braak stage (Karikari *et al.*, 2020), whilst NFL is not (Thijssen *et al.*, 2020). Interestingly, a decreased ratio of Ptau_181_/NFL was able to distinguish FTLD from Alzheimer’s disease, likely as a result of non-Aβ driven (and therefore not involving P-tau_181_) neuronal damage (Janelidze *et al.*, 2020).

## Conclusion

The studies of de Wolf *et al.* and Lopez *et al.* have contributed important longitudinal data to the emerging picture of blood-based biomarkers for neurodegenerative disease. With further validation, blood biomarker analysis has the potential to synergise with polygenic risk score screening and open up a paradigm where one might go to a general practitioner, and receive multiple biomarker measurements monitored longitudinally. This could guide pre-emptive therapy aimed at managing concentrations relative to an individual’s own normal.

## Funding

H.Z. is a Wallenberg Scholar supported by grants from the Swedish Research Council (#2018-02532), the European Research Council (#681712), the Swedish state under the agreement between the Swedish government and the County Councils, the ALF-agreement (#ALFGBG-720931), the Alzheimer Drug Discovery Foundation (ADDF), USA (#201809-2016862) and the UK Dementia Research Institute at UCL. J.T. is supported by the UK Dementia Research Institute which receives its funding from DRI Ltd, funded by the Centre for Discovery Brain Sciences and the UK Medical Research Council, Alzheimer’s Society, and Alzheimer’s Research UK and the European Research Council (ERC) under the European Union’s Horizon 2020 research and innovation programme (Grant Agreement No. 681181).
